# Phytomedicines and Nutraceuticals: Alternative Therapeutics for Sickle Cell Anemia

**DOI:** 10.1155/2013/269659

**Published:** 2013-02-14

**Authors:** Ngozi Awa Imaga

**Affiliations:** Department of Biochemistry, Faculty of Basic Medical Sciences, College of Medicine, University of Lagos, PMB 12003, Idi-Araba, Lagos, Nigeria

## Abstract

Sickle cell anemia is a genetically inherited disease in which the “SS” individual possesses an abnormal beta globin gene. A single base substitution in the gene encoding the human **β**-globin subunit results in replacement of **β**6 glutamic acid by valine, leading to the devastating clinical manifestations of sickle cell disease. This substitution causes drastic reduction in the solubility of sickle cell hemoglobin (HbS) when deoxygenated. Under these conditions, the HbS molecules polymerize to form long crystalline intracellular mass of fibers which are responsible for the deformation of the biconcave disc shaped erythrocyte into a sickle shape. First-line clinical management of sickle cell anemia include, use of hydroxyurea, folic acid, amino acids supplementation, penicillinprophylaxis, and antimalarial prophylaxis to manage the condition and blood transfusions to stabilize the patient's hemoglobin level. These are quite expensive and have attendant risk factors. However, a bright ray of hope involving research into antisickling properties of medicinal plants has been rewarding. This alternative therapy using phytomedicines has proven to not only reduce crisis but also reverse sickling (*in vitro*). The immense benefits of phytomedicines and nutraceuticals used in the management of sickle cell anemia are discussed in this paper.

## 1. Introduction

### 1.1. Hemoglobinopathies: Sickle Cell Anemia

Sickle cell disorder (SCD) is a group of hereditary illnesses affecting the red cell hemoglobin [1]. Various types of these disorders exist: including sickle thalassaemia and sickle cell anemia (HbSS), also known as drepanocytosis. The disease is most prevalent in the black race, but it is also known in other races surrounding the Mediterranean and in India [2]. 

**Figure 1:**
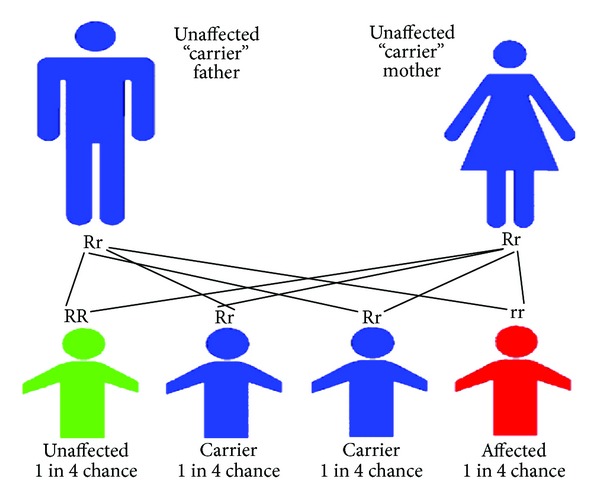
Sickle cell disorder inheritance pattern.

Parents who possess heterozygous genotypes (HbAS) are sickle cell carriers and their offspring have a 1 in 4 chance of having a homozygous sickle genotype (HbSS) or a homozygous normal genotype (HbAA) as depicted in Figure 1. 

**Figure 2:**
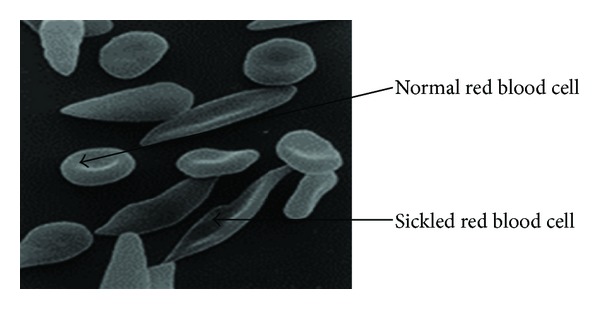
Normal and sickled red blood cells [1].

Sickle cell disease was first recognized as a hematological disorder by Herrick in 1910 and its molecular pathology was established in 1949 by Linus Pauling. Molecular research traces its origin to the study of abnormal hemoglobin and the mechanisms by which a single base substitution in the gene encoding the human *β*-globin subunit, with the resulting replacement of *β*6 glutamic acid by valine, leads to the devastating clinical manifestations of sickle cell disease [1]. This substitution causes a drastic reduction in the solubility of sickle cell hemoglobin (HbS) when deoxygenated. Under these conditions, the HbS molecules polymerize to form intracellular fibers which are responsible for the deformation of the biconcave disc shaped erythrocyte into a sickle shape [3]. The normal and sickled red blood cells are shown in Figure 2.

The ailment is characterized by premature breakdown of the red blood cells causing constant anemia and occlusion of small blood vessels leading to excruciating body pains and other manifestations. The disease stems from inadequate oxygen transport by red blood cells. *In vivo*, sickled erythrocytes tend to block capillaries, causing stasis, and thereby starve organs of both nutrients and oxygen and eventually cause hypofunction or complete tissue destruction [1]. Sickle cell incidence has been closely linked to malaria incidence in tropical areas like Nigeria. These SS persons are least fit for survival in a hostile malaria environment and survival rates are particularly low in childhood.

## 2. Approach to Therapy

There are several compounds such as amino acids, which prevent sickling by affecting the erythrocyte membrane, causing an increase in the cell volume of the erythrocyte and thus reducing the intracellular hemoglobin concentration below its minimum gelling concentration [4–8]. The most popular approach to prevent or reverse sickling *in vitro* and *in vivo* is to employ compounds or techniques which directly affect the hemoglobin (Hb) molecule.

A characteristic property of the gelation of deoxy-HbS is the existence of a delay time prior to polymerization of deoxy-HbS molecules [9]. A drug that prolongs the delay time prior to polymerization might be of therapeutic value in SCD, because a longer delay time decreases the probability of SS cell sickling. Reported antisickling/antidrepanocytary agents in this group include a formulated phytomedicine, Niprisan (Nix-0699), a chemical compound 5-hydroxymethyl-2-furfural [5HMF], and MX-1520 (a prodrug of a food additive, Vanillin) which modify intracellular sickled hemoglobin and inhibit sickling of red blood cells [10–13].

Presently, first-line clinical management of sickle cell anemia includes use of hydroxyurea, folic acid and amino acids supplementation (as nutritional supplements), penicillin prophylaxis (helps prevent infection), and antimalarial prophylaxis (helps prevent malaria attack), for example, paludrine in varying doses in childhood, adulthood, and pregnancy. The faulty “S” gene is not eradicated in treatment; rather the condition is managed and synthesis of red blood cells induced to stabilize the patient's hemoglobin level. Further management and treatment of this disorder with compounds or techniques which directly affect the hemoglobin [Hb] molecule (e.g., hydroxyurea, bone marrow transplantation, and blood transfusion) are very expensive and out of reach of the masses and besides expose the patient to mutagenicity, iron overload, and other fatal risks [14–17]. Monthly blood transfusion lowers the proportion of sickling cells to <30%, but it is stopped at 18 years of age. Others recommend transfusion of stroke patients (from cerebrovaso occlusion) for an indefinite period of time in view of the high recurrence risk (of the stroke). However, there is a predictable complication of long-term therapy because the anemia is not an iron deficiency condition, rather a hemolytic type. Therefore the patient already has the required iron concentration in the blood and may run the risk of iron-overload. Bone marrow transplantation is a more definitive treatment [15]. Another angle for drug relief adduces the reason for the stickiness of SS red blood cells to be due to the secretion of thrombospondin, a cell surface protein [14].

In summary these are the various approaches to sickle cell disease therapy

- Clinical/medical/pharmacological:
  - blood transfusion, bone marrow transplantation.
  - chemotherapy: hydroxyurea, which increases HbF (an antagonist of HbS) stimulation, nitric oxide gas inhalation.
  - confers only symptomatic relief/maintenance of patient.
  - anti-inflammatory (for pain crisis).
  - antimalarial and antibacterial drugs (paludrine and penicillin).
- Nutritional:
  - multivitamin supplements, proper diet, calorie and protein intake, Vanillin.
- Phytomedicines/phytotherapy. Phytomedicines and naturally occurring antisickling agents:
  - Niprisan with *Piper guineense*, *Pterocarpus osun*, *Eugenia caryophyllum*, and *Sorghum bicolor* as components, Ciklavit (*Cajanus cajan* as base), and hydroxybenzoic acids are used in SCD management [13, 18].

## 3. Antisickling Agents

Synthetic (otherwise called, orthodox) medicines developed so far for sickle cell management focus on symptomatic relief of pain and crisis alleviation. Examples of such drugs are zinc, piracetam (which aim to prevent sickle cell crisis by reducing red blood cell dehydration), PP-188 (Purified Poloxamer 188) blood drug (which reduces the viscosity of RBC's), and nitric oxide gas [19–21]. 

Research into antisickling properties of medicinal plants has been rewarding. This alternative therapy using phytomedicines has proven to not only reduce crisis but also reverse sickling (*in vitro*). Examples of these herbal drugs are Niprisan (renamed Nicosan) with *Piper guineense, Pterocapus osun, Eugenia caryophyllum, *and* Sorghum bicolor *as components; Ciklavit (*Cajanus cajan *seed extract as base), Aqueous extracts of *Zanthoxylum zanthoxyloides* roots, Ajawaron HF complex with *Cissus populnea* as main component, Aqueous and alcoholic extracts of *Terminalia catappa* leaves; *Carica papaya* unripe fruit and dried leaf extracts.

*Zanthoxylum zanthoxyloides* (otherwise called *Fagara*, orin-ata) roots have been analyzed for antiprotease and membrane stabilizing activity using a modified osmotic fragility technique to analyze membrane stabilization action [22]. It has been discovered that the antisickling (and anti-inflammatory) action of *Fagara* was due to its o-hydroxybenzoic acid constituent [23]. According to literature, these already documented herbs and compounds, for example, *Cajan*, *Fagara*, Niprisan, and Ciklavit are all still in the research stage and some have passed through clinical trials and health care safety standardizations and have been approved for use [4, 18, 24–29]. While ascertaining the efficacy of these drugs, their safety in humans is also important for survival. The mode of action of these herbal drugs is of particular interest. The possible mechanism of action of phenylalanine, an amino acid reported to have antisickling effect, has been adduced, indicating the role of several transport systems [3, 30, 31]. 

Aqueous and ethanolic extracts of several phytomedicines have been evaluated for significant *in vitro* antisickling activity. Recent studies support the claims of the traditional healers and suggest a possible correlation between the chemical composition of these plants and their uses in traditional medicine [32]*. Z. zanthoxyloides* has shown drepanocyte (sickling) reversibility, appreciable increase in hemoglobin gelling time, and improved rheological properties of drepanocytary blood [33]. Antisickling properties of amino acids have been recognized much earlier; of all the amino acids reported, phenylalanine was shown to be most active [6–8]. The mode of transport and possible mechanism of action of some amino acid benzyl esters, for example, L-phenylalanine benzyl ester (Phe-Bz), an aromatic compound, an antisickling agent was found to be effective at a low concentration and is therefore a potential therapeutic agent for the treatment of sickle cell disease [3].

The antisickling effect of cetiedil [alpha-cyclohexyl-3-thiopheneacetic acid 2-(hexahydro-1 Hazepin-1-yl) ethyl ester] has been reported [34]. Antisickling effect was achieved regardless of whether cetiedil was added before or after deoxygenation. The minimal gelling concentration of deoxy HbS was increased by less than 10% in the presence of cetiedil concentration and the oxygen equilibrium curves of HbS were not significantly affected. Erythrocytes treated with high concentrations of cetiedil were swollen and became spheroidal. It was concluded that the antisickling effect of cetiedil might be due to an effect on erythrocyte membranes. The hemoglobin (Hb) molecule has a high affinity for most substrates that reverse the sickling phenomenon [5]. The configuration of the region of Hb where antisickling agents bind has been determined, suggesting that the rate constant *k* is dependent upon the rate at which the substrate is transported across the membrane since the rate of combination with hemoglobin is very fast.

Another scope of work on antisickling agents with the focus on nutrition found that the concentrations of ascorbic acid and alpha-tocopherol were significantly depressed while that of retinol was slightly reduced in subjects tested. The depletion in the levels of the antioxidant vitamins A, C, and E may account for some of the observed manifestations of sickle cell anemia, such as increased susceptibility to infection and hemolysis [35]. Vitamin B_12_ levels have been observed to be diminished in patients with severe sickle cell disease. Patients with low vitamin B_12_ achieved a significant symptomatic improvement when treated with vitamin B_12_, 1 mg intramuscularly weekly for 12 weeks. It was concluded that many patients with severe sickle cell disease may suffer from unrecognized vitamin B_12_ deficiency [36].

Research on antioxidant status and susceptibility of sickled erythrocytes to oxidative and osmotic stress has been reported using a range of diluted saline-phosphate buffer in a typical osmotic fragility test to determine osmotic stress/membrane integrity and AAPH (a peroxyl radical generator) to induce hemolysis with oxygenated and deoxygenated RBCs for oxidative stress analysis. It was discovered that though there are differences in antioxidant status between sickled and normal RBCs, these differences did not appear to be responsible for the observed difference in susceptibility to oxidative or osmotic stress-induced hemolysis [37].

The inhibition of erythrocyte membrane ATPases with antisickling and anesthetic substances and ionophoric antibiotics has been studied, in the light of the partition coefficient of these drugs in erythrocyte membranes, the changes they induce in the permeability properties of erythrocytes, and the subsequent effect of procaine on sickling of erythrocytes and their potential interaction with specific membrane components. In general, the drugs were found to inhibit both types of enzymic activities but with varying degrees of efficacy. (Ca^2+^-Mg^2+^)-ATPase was more sensitive to the lipophilic anesthetics and (Na^+^-K^+^)-ATPase to the ionophoric antibiotic, Amphotericin B [38].

Oral magnesium supplementation reduces the number of dense erythrocytes and improves the erythrocyte membrane transport abnormalities of patients with sickle cell disease. Children with SCD are reported to demonstrate or exhibit normal serum magnesium level with accompanying hyperphosphataemia and hypocalcaemia [39]. They have also been observed to have decreased height and weight when compared with their peers. Although exact reasons for poor growth were not established, increased calorie and protein needs and deficiencies in zinc, folic acid, and vitamins A, C, and E were adduced to be the factors responsible [40, 41]. It has been suggested that the nutrient intake of patients with sickle cell disease is often inadequate and the study suggests that education of patients with SCD should focus on specific nutrient needs, with proper distribution of dietary intake among the food groups, ways to provide nutritious meals on a limited income, and methods for increasing calorie and protein intake. Patients with SCD that have adequate vitamin B_6_ and B_12_ status, but elevated plasma homocysteine levels with indicated suboptimal folate status, especially pediatric sickle cell patients, may benefit from folate supplementation to reduce their high risk for endothelial damage [42].

A potential nutritional approach for the molecular disease SCD found that from both *in vitro* and pilot clinical trials, a “cocktail” of aged garlic extract, vitamin C, and vitamin E proved beneficial to patients. Ascorbic acid is important in SCA because significant oxidative stress occurs in the disease and its role as an antioxidant is very beneficial [43].

Ascorbate levels in red blood cells and urine in patients with sickle cell anemia have been analyzed [44] and it was reported that

1. ascorbate is present in sickled red blood cell (SRBC), most likely due to ascorbate recycling, despite increased free-radical generation;
2. there is increase in renal excretion, which may contribute to the low plasma levels of ascorbate;
3. the presence of ample ascorbate in sickled red blood cells (SRBCs) and decreased plasma ascorbate suggests that ascorbate movement across the SRBC membrane may be different from normal red blood cell.

The effect of vitamin C on arterial blood pressure, irreversibly sickled cells (ISCs), and osmotic fragility in sickle cell anemic subjects also suggests a potential benefit of vitamin C supplementation to sickle cell anemia subjects because vitamins A, C, and E supplementation was shown to decrease arterial blood pressure, % ISCs (irreversibly sickled cells), and MCHC (mean corpuscular hemoglobin concentration) but increased Hb (hemoglobin) and PCV (packed cell volume) [45, 46].

The first widely accepted herbal formulation Niprisan (now Nicosan) produced by Wambebe of NIPRID, Abuja, Nigeria, was analyzed *in vivo* using transgenic mice under acute severe hypoxic conditions using a series of innovative analyses and tests and found to be very effective in reversing and preventing sickling [9, 12]. The kinetics of reversal of presickled erythrocytes by aqueous extract of *C. cajan* seeds was reported [47] as well as the antisickling properties of *Parquetina nigrescens* and a Nigerian herbal formula, Ajawaron HF, using the method of sodium metabisulphite-inhibition of sickling for the analysis [26, 48].

The antisickling effects of MX-1520, a prodrug of vanillin, have been analyzed using rodents *in vivo*. This prodrug was produced because vanillin rapidly decomposed in the upper digestive tract and so was ineffective when taken orally in its original form [11]. A naturally occurring aromatic aldehyde, 5-hydroxymethyl-2-furfural (5HMF), was found to modify intracellular sickled hemoglobin and to inhibit sickling of red blood cells. This aldehyde unlike previous ones was found to be bioavailable (i.e., did not get decomposed in the digestive tract, but was found in appreciable amounts in the blood stream) [10].

More research on antisickling agents have evolved since then especially in Nigerian universities, with the emphasis on antisickling action of extracts of phytomedicines and isolated antisickling agents contained in these phytomedicines. For example, the antisickling activity of *Carica papaya* unripe fruit extracts [49–52] and *Carica papaya* dried leaf extract [53, 54] have been reported.

## 4. Current Trends in Alternative Herbal Treatment of Sickle Cell Anemia

**Scheme 1:**
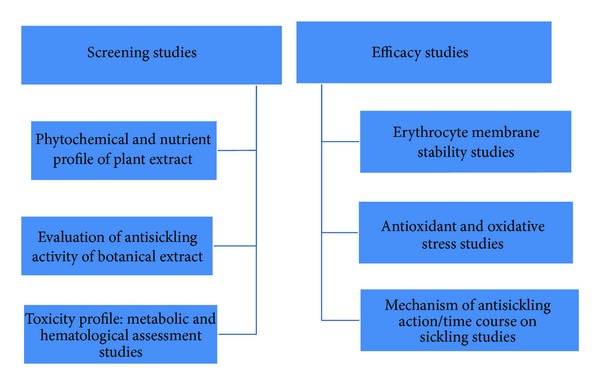
A scheme of research showing a flowchart of methodologies and research phases used in our laboratories to screen and ascertain the efficacy of antisickling phytomedicines.

It is acknowledged worldwide that traditional medicine can be explored and exploited to be used alongside synthetic pharmaceutical products for enhanced health management. Due to the high mortality rate of sickle cell patients, especially in children, and since chemotherapy has its adverse effects, there is need for rational drug development that must embrace not only synthetic drugs but also natural products (phytomedicines/herbal drugs), naturally occurring antisickling agents which can be obtained from our vast forest resources and can be used to effectively manage the sickle cell patient and treat the anemic condition accompanying this disorder. Attempts to find alternative, cheaper, and less toxic therapies led to the scientific discovery of antisickling properties of some medicinal plants such as *Cajanus cajan* seeds, *Zanthoxylum zanthoxyloides (Fagara)* root, *Carica papaya* unripe fruit, and also *Parquetina nigrescens* whole plant extracts which boost blood volume—all these are locally used by traditional healers in Nigeria for diverse herbal remedies [23, 31, 48–52]. Medicinal plants are parts of a plant or the whole plant that possess healing properties and unlike orthodox (synthetic) medicines, which may have adverse side effects, medicinal plant formulations are considerably cheaper and safer to use. In a previous review, selected medicinal plants with antisickling properties which are currently in use for the management of sickle cell anemia were highlighted and their methods of extraction, the various methods of analyzing herbal extracts for antisickling activity via efficacy tests and analyses and research findings were also discussed [55]. Since then, more research studies have continued in our laboratories and some of the findings are summarized here (Scheme 1).

### 4.1. *Carica papaya* Dried Leaf Extract

*Carica papaya* is a member of the Caricaceae family, native to Nigeria and Central America, and is medicinal plant used as an alternative therapeutic agent for sickle cell anemia. The correlations between the chemistry and pharmacology of *Carica papaya* leaves have been reported. Phenolic compounds have been found in papaya leaves [56]. The presence of such compounds could partially explain the pharmacological properties of this plant and demonstrate its importance in alimentation and daily intake. Phenolic compounds are important components in vegetable foods, infusions, and teas for their beneficial effects on human health. 

Methanol extract of papaya leaves has been analyzed and important polar compounds (secondary metabolites) were identified and quantified using gas chromatography-mass spectrometry (GC-MS) in the selected ion-monitoring (SIM) mode. 5,7-Dimethoxycoumarin and polar molecules such as protocatechuic acid, *p*-coumaric acid, chlorogenic acid, kempferol, and quercetin were detected and identified in qualitative analysis. Quantitative analysis showed the presence of phenolic acids as the main compound, while chlorogenic acid was found in trace amounts, compared to the flavonoids and coumarin compounds [56].

### 4.2. Effects of Papaya Leaf Extracts on Sickling

Many phytomedicines have been identified as potential antisickling agents, stemming from reported usage as ethnomedicines by the local folk. *Carica papaya *dried leaves have been indicated in sickle cell anemia management by local indigenous folk and in recent scientific research. A particular research examined methanolic leaf extracts of *Carica papaya* L. (Caricaceae) for possible *in vitro* antisickling and membrane-stabilizing activities involving the use of positive (p-hydroxybenzoic acid 5 *μ*g/mL) and negative (normal saline) controls for the antisickling experiments and osmotic fragility test on Hb^ss^ red blood cells obtained from noncrisis state sickle cell patients. Fragiliograms indicated that the plant extract reduced hemolysis and protected erythrocyte membrane integrity under osmotic stress conditions. Pretreatment of SS cell suspensions with *Carica papaya* leaf extract inhibited formation of sickle cells under severe hypoxia, with only 0–5% sickle cells at 40 mins compared with untreated SS cell suspensions which had over 60% sickle cells. These results indicate the feasibility of *Carica papaya* as an attractive potential candidate for SCD therapy [53].

In another research, dried *C. papaya *leaves were extracted using the soxhlet extraction method with 5 different solvents to give five different fractions, namely, hexane, chloroform, ethyl acetate, butanol, and water. The research examined the crude extract and the various leaf extract fractions of *C. papaya *L. (Caricaceae) for possible *in vitro *antisickling activities on Hb^ss^ red blood cells obtained from noncrisis state sickle cell patients involving the use of positive (phydroxybenzoic acid 5 *μ*g/mL) and negative (normal saline) controls for the antisickling experiments. Pretreatment of SS cell suspensions with *C. papaya *leaf extract and fractions all inhibited formation of sickle cells under severe hypoxia at varying degrees, with only 0–5% sickle cells in the crude extract at 60 min compared with untreated SS cell suspensions which had over 80% sickle cells. Analysis of two different concentrations of *C. papaya *crude extract (10 and 5 mg/mL) showed the 10 mg/mL extract as the concentration with highest antisickling effect. Butanol extract showed the highest antisickling activity at 10 mg/mL concentration, while the ethyl acetate extract had the highest antisickling activity at 5 mg/mL concentration. These results further indicate the possibility of *C. papaya *leaf extract as potential phytotherapy for sickle cell anemia [54]. 

### 4.3. Antioxidant Effects of Papaya Leaf Extracts, *Cajanus cajan* Seed Extract, *Fagara zanthoxyloides* Root Extract, and *Parquetina nigrescens* Plant Extract on the Erythrocyte

**Figure 3:**
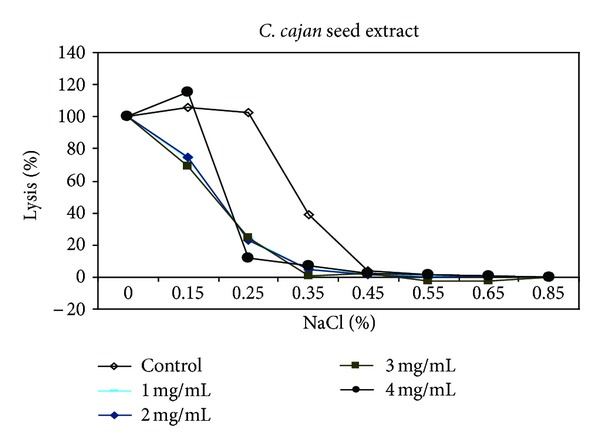
Osmotic fragiliograms after supplementation with various concentrations of *C. cajan* seed extract.

**Figure 4:**
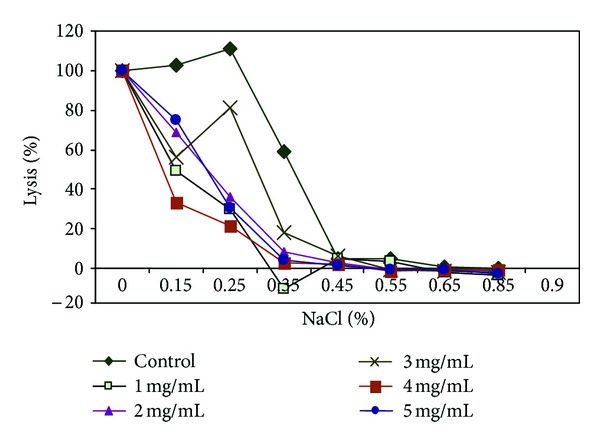
Osmotic fragiliograms after supplementation with various concentrations of *Fagara* root extract.

**Figure 5:**
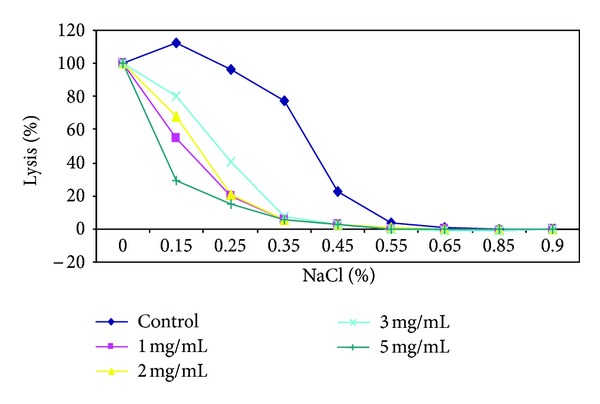
Osmotic fragiliograms after supplementation with various concentrations of *Parquetina nigrescens *plant extract.

**Figure 6:**
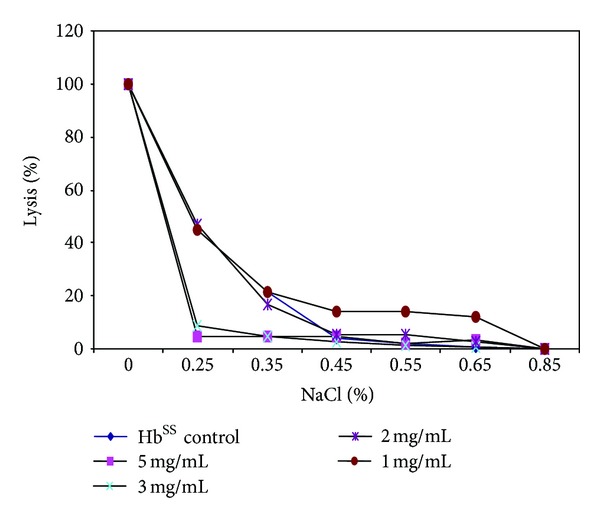
Osmotic fragiliogram after supplementation with various concentrations of *Carica papaya* extract.

In demonstration of the ability of *Carica papaya* leaf extract to confer protective properties on the erythrocyte membrane, the effect of varied concentrations of the herbal extracts on erythrocyte membranes was analyzed using the osmotic fragility test, which revealed appreciable membrane-stabilizing (protective) effects of the herbs and their inhibitory action on hemolysis of red blood cells (Figures 3, 4, 5, and 6). The resistance of the erythrocytes can be measured by subjecting them to the action of various harmful agents. Red blood cells suspended in hypotonic salt (NaCl) solution take up water, swell, and become spheroidal and more fragile, and eventually burst. The increased fragility, which leads to lysis, is inversely proportional to the concentration of NaCl and directly proportional to the thickness of the red blood cell [57]. An increase in osmotic fragility is equivalent to a decrease in osmotic resistance. Rounded cells lyse at relatively high salt concentrations. The osmotic fragility test measures accurately how nearly spherical red cells are. Increased osmotic fragility or decreased resistance means spherocytosis (found in hereditary spherocytosis, hemolytic anemia). Diminished osmotic fragility or increased resistance means excessive flatness of red cells (sickle cell anemia, jaundice, and thalassemia). In a study [53] most of the cells supplemented with papaya extract were still rounded after incubating in salt solutions. This observed inhibition/reduction in RBC lysis after treatment with the *C. papaya* extract is indicative of protective properties of the extracts on the RBC membrane, thus helping to maintain membrane integrity through membrane stabilization.

Sickle erythrocytes have been reported to have a distorted volume-to-surface ratio when compared to normal erythrocytes [45] and so a shift to the left in the osmotic fragiliograms suggests a higher osmotic resistance for most sickle cells. This shift was observed in the study, showing that the extract was able to protect the integrity of the erythrocyte membrane, increase its resistance to osmotic stress/lysis, and thus reduce membrane fragility. From these erythrocyte studies, one can infer that aqueous extract of *Carica papaya* reduced hemolysis and conferred some protective effect on erythrocyte membrane.

Active constituents of medicinal plants and naturally occurring compounds, known as antisickling agents, which improve the health of sickle cell individuals are rich in aromatic amino acids, phenolic compounds, and antioxidant nutrients [58] which are thought to be responsible for their observed antisickling action. A herbal preparation of *Cajanus cajan* was found to contain phenylalanine, carjaminose, and hydroxybenzoic acid as active constituents and are thought to be the reason for its antisickling effect [47]. Folk medicine reportedly uses *Parquetina nigrescens* L. (Asclepiadaceae) as a herbal remedy for the management of sickle cell anemia. A study was carried out to screen the leaves and stem of *Parquetina nigrescens* for antisickling activity, erythrocyte membrane-stabilizing effects, and any end organ toxicity. Percentage reversal and inhibition of sickling parameters were analyzed on presickled Hb^SS^ blood cell suspensions using sodium metabisulphite solution as inducer and 5 mg/mL parahydroxybenzoic acid and normal saline as positive and negative controls, respectively. Effects of the plant extracts on the erythrocyte were assessed using osmotic fragility and the toxicity profile done via lethal dose LD_50_ and subacute toxicity studies on graded concentrations of extract. Results showed that *Parquetina nigrescens* has appreciable antisickling activity, has no toxic effect when administered at low concentrations, and protects the integrity of the erythrocyte membrane as evidenced in the fragiliogram by the reduction in hemolysis of the Hb^ss^  cells [59]. The presence of alkaloids and flavonoid glycosides could also act as an adjuvant that enhances the activity of the components actually responsible for the membrane protection effect noticed in the fragiliograms. From reported findings, one can appreciate the antioxidant properties of these phytomedicines and their role in maintaining the integrity of red blood cells and subsequently improving the quality of life in individuals with sickle cell anemia.

**Figure 7:**
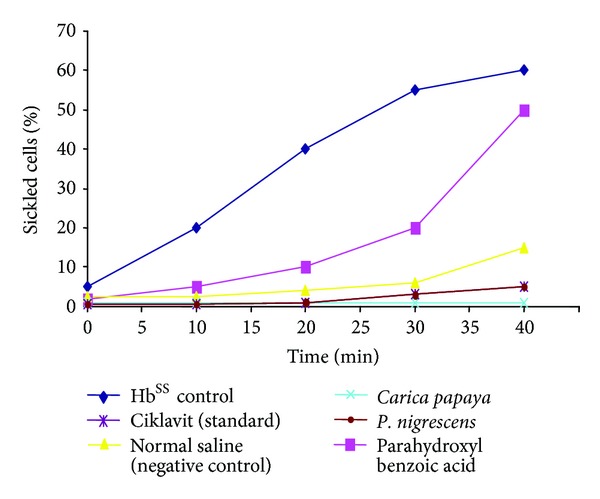
Comparison of antisickling activities of phytomedicines.

**Figure 8:**
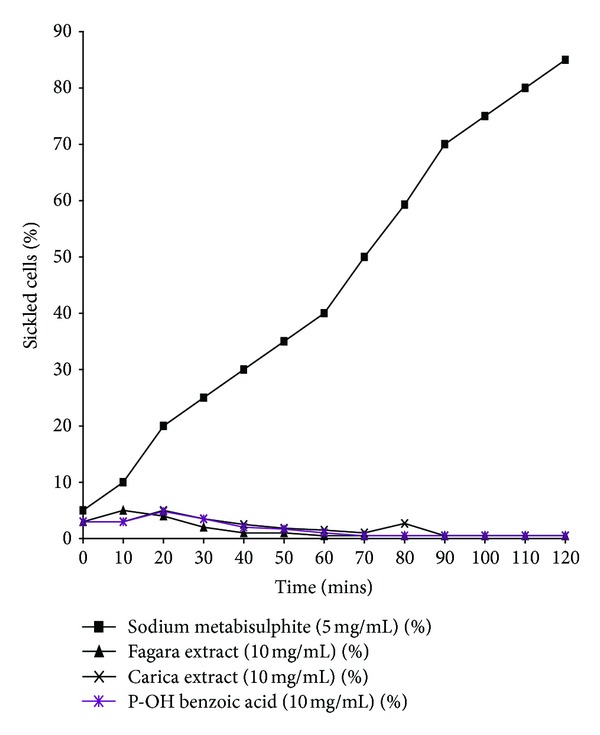
Time course of phytomedicines on Sickling.

Various works have identified a number of herbal applications that have ameliorating effects on sickle cell disorders (Figures 7 and 8). The antisickling activities of dried *Carica papaya *leaves and roots of *Fagara zanthoxyloides *were investigated in a study to determine the antioxidant properties of the plant extracts and their effects on homozygous sickle cell (SS) erythrocytes *in vitro*. The antisickling activities of both extracts were determined as well as analyses of hematological parameters, hemolysis of SS cells, and formation of membrane-associated denatured hemoglobin (MADH) used to measure the effects of plant extracts on the erythrocyte. Folin-C total phenol and beta-carotene methods of assay were used to determine antioxidant activity, while the effect of plant extracts on oxidative stress was measured by assaying for superoxide dismutase, catalase, glutathione transferase levels, and lipid peroxidation. Results confirmed the potent antisickling activity of both plants. The levels of the oxidative stress enzymes superoxide dismutase (SOD), catalase (CAT) and glutathione (GST) and lipid peroxidation were reduced after blood samples had been incubated with the extracts. The extracts therefore protected membrane integrity resulting in a reduction of red blood cells (RBCs) hemolysis without met-hemoglobin formation. It was concluded that both plant extracts possess potent antioxidant activity which may be responsible for their observed antisickling action [60]. 

Methanol extracts of herbs hitherto reported to have antisickling activity namely, *Carica papaya* leaf extract, *Fagara zanthoxyloides* root extract, *Cajanus cajan* seed extract, and *Parquetina nigrescens* leaf extract were evaluated in another study. An assessment of their antioxidation potential was determined by assaying for their phytochemical constituents, total phenol content, scavenging activity on DPPH, and total antioxidant status via the ferric thiocyanate method. The extracts had similar phytochemical constituents and exhibited high scavenging activity compared to gallic acid and ascorbic acid standards due to their relatively high total phenol content. These findings suggest that *Carica papaya* leaf extract, *Fagara zanthoxyloides* root extract, *Cajanus cajan* seed extract, and *Parquetina nigrescens* leaf extract are endowed with antioxidant phytochemicals which may act singly or synergistically to potentiate the antisickling action of the plants [61].

## 5. Correlation between Oxidative Stress, Antioxidants, and Sickling

### 5.1. Oxidative Stress and Sickling

Oxidative stress is caused by an imbalance between the production of reactive oxygen species and a biological system's ability to readily detoxify the reactive intermediates or easily repair the resulting damage. It arises when the cellular generation of reactive oxygen species (ROS) overwhelms the antioxidant defense system [62]. Oxidative stress is a large increase in the cellular reduction potential or a large decrease in the reducing capacity of cellular redox couples such as glutathione. Oxidative stress challenges often arise from sources such as radiation, metabolism of xenobiotics, and challenges to the immune system or abnormal functions [63]. 

Many health hazards such as atherosclerosis, cancer, Parkinson's disease, and Alzheimer's disease have been associated with oxidative stress. Amongst other associated diseases of oxidative stress are sickle cell diseases such as sickle cell anemia. Sickle cell crisis (the fall out of the sickling phenomenon) usually starts with inflammation of joints, which is mostly as a result of oxidative stress affected erythrocytes (RBCs). A very destructive aspect of oxidative stress is its production of reactive oxygen species, which include free radicals and peroxides [63]. 

However, ROS can sometimes be beneficial as they can be used by the immune system as a way to attack and kill pathogens and also as a form of cell signaling. Most of these oxygen-derived species are produced at low levels by normal aerobic metabolism and the damage they cause to cells is constantly repaired. However, under the severe levels of oxidative stress that cause necrosis, the damage causes ATP depletion, preventing controlled apoptotic death and causing the cell to fall apart [63].

### 5.2. Antioxidants: Vitamins and Enzymes

Antioxidants are literarily known as “scavengers” or “moppers” of free radicals in an organic entity. They scavenge for free radicals and, consequently, are a very special group of nutritional supplements [64]. The term antioxidant (also “antioxygen”) was originally used to refer specifically to a chemical that prevented the consumption of oxygen. In the late 19th and early 20th centuries, extensive study was devoted to the uses of antioxidants in important industrial processes, such as the prevention of metal corrosion, the vulcanization of rubber, and the polymerization of fuels in the fueling of internal combustion engines. However, early research on the role of antioxidants focused on their use in preventing the oxidation of unsaturated fats, which causes rancidity. Then, antioxidant activity could be measured simply, by placing the fat in a closed container with oxygen and measuring the rate of oxygen consumption. Yet, it was the identification of *β*-carotene (precursor of vitamin A), vitamin C (ascorbic acid), and vitamin E (*α*-tocopherol) as antioxidants that revolutionized the field and led to the realization of the importance of antioxidants in the biochemistry of living organisms.

Antioxidants and their mechanisms of action were first explored when it was recognized that a substance with antioxidative activity is likely to be one that is itself readily oxidized. Further research into how vitamin E prevents the process of lipid peroxidation led to the identification of antioxidants as reducing agents that prevent oxidative reactions, often by scavenging reactive oxygen species (ROS) before they can damage cells. 

Among the numerous antioxidants available, flavonoids are naturally occurring phenolic compounds in plants. In fact, the majority of antioxidants, both natural and synthetic, are phenolic compounds [65]. Vitamin C, beta-carotene, and vitamin E are all powerful natural antioxidants. Other natural antioxidants, which have been less well characterized, are the flavonoids and anthocyanins. These are all phenols and several thousand different structural variants are found in nature. It has been estimated that the total intake of these compounds in the typical diet is close to 1000 mg per day [65], dwarfing the antioxidant content of antioxidant vitamins in the typical diet. Due to their remarkable importance, antioxidants have been the focus of considerable research and the antioxidant properties of several plants *in vivo* and *in vitro* have been shown to be of great advantage to nutrition today.

For sickle cell disease (SCD), the study of antioxidants especially in various antisickling agents is of great importance because different antisickling agents have different degrees of effect. Antioxidants (scavengers of free radicals) are believed to be major components of these antisickling agents that add to their potential [37]. Thus, it is believed that the higher the antioxidant property of an antisickling agent, the higher its possible antisickling effect, as this enables it to reduce oxidative stress that contributes to sickle cell crisis. 

In a reported research [60], aqueous extracts of *Carica papaya* and *Zanthoxylum zanthoxyloides* showed high total antioxidant properties (via *β*-carotene bleaching assay) and higher phenolic properties than garlic acid. This might explain why decoctions of these plants (used locally) over the years give relief to various oxidative stress associated diseases. The levels of the oxidative stress enzymes (SOD, CAT, and GST) were reduced after blood samples had been incubated with papaya extracts. These enzymes are excreted from the cell during cell damage. Lipid peroxidation, measured indirectly by the percentage of malonaldehyde (MDA) inhibited by plant extract, was also reduced by papaya extract. These findings further confirm the antioxidant activity inherent in the plant extract. Hb^SS^ individuals already in distress during oxidative stress-induced RBC membrane lysis do not need this situation aggravated by a plant extract that causes more oxidative stress to the erythrocyte membrane. Low levels of the oxidative stress enzymes were observed and indicate that papaya extracts can quickly mop up free radicals produced during sickle cell crisis and thus help preserve the integrity of the membrane, along with its inherent nutrients and the glutathione synergistic effect.

## 6. Herbal Preparations Already in Use as Government-Approved Phytomedicines and Nutraceuticals for Sickle Cell Anemia Management

### 6.1. Nicosan

Nicosan (formerly known as Niprisan), an antisickling phytomedicine, is reported to inhibit the polymerization of the hemoglobin S. As reported earlier, it is a cocktail of four medicinal plants, *Piper guineense, Pterocapus osun, Eugenia caryophyllum, *and* Sorghum bicolor*, as components and is currently being marketed in Nigeria in encapsulated 250 mg/350 mg doses for a once-daily administration. In a research, the biochemical effects of drug-drug interaction of Ciprofloxacin, a wide spectrum antibiotic, coadministered with Nicosan were examined using standard methods for biochemical, hematological, and antioxidant assays. Findings showed that the presence of Nicosan had a palliative effect on the oxidative free radicals produced as a result of the antibiotic administration [66]. 

### 6.2. Ciklavit

Ciklavit is a plant extract preparation available for the management of the sickle cell anemia condition. It contains primarily extracts of the plant *Cajanus Cajan*, proteins (essential amino acids), vitamins such as vitamin C (ascorbic acid), and minerals such as zinc. Ciklavit (*Cajanus cajan *extract) has been reported to have antisickling properties and to improve well being of sicklers. The study also revealed that the antisickling effect of Ciklavit may not probably be through nitric oxide generation or arginase inhibition, since there were no appreciable changes in these parameters. Earlier reports of the antisickling constituent of *Cajanus cajan *suggested cajaminose [67], phenylalanine, and hydroxybenzoic acid [30]. Phytochemical studies on the aqueous extract confirm the presence of phenylalanine and several other amino acids and phenolic compounds and tannins. The antisickling properties of amino acids in *in vitro* studies have been recognized much earlier. Of all the amino acids reported, L-phenylalanine, found to have antigelling effects, was shown to be most active [7]. The role played by other components in Ciklavit (besides *Cajanus cajan*) is basically nutritional. Blood levels of several vitamins and minerals are often low in individuals with sickle cell disease, including vitamin A and carotenoids, vitamin B6, vitamin C, vitamin E, magnesium, and zinc [44, 68–73]. These deficiencies cause a significant depreciation in blood-antioxidant status in these patients [74] and the resulting oxidative stress may precipitate vasoclusion-related acute chest syndrome [75]. Studies indicate that vitamin-mineral supplements of certain nutrients (vitamins C and E, zinc, and magnesium) or treatment with a combination of high-dose antioxidants can reduce the percentage of irreversibly sickled cells [40, 43, 71, 76, 77]. Zinc sulphate appears to help reduce red blood cell dehydration. Important studies indicate that it helps prevent sickle cell crises and reduce pain and life-threatening complications. A study on children with sickle cell suggested that supplements may help improve growth and weight gain. It may also boost the immune system and help protect against bacterial infections. Zinc deficiency is a common nutritional problem in sickle cell disease, so supplements may be important. Magnesium protects against potassium and water loss in sickle cells [19, 72, 73]. In view of these findings, it can be concluded that Ciklavit may cause a reduction in bone pains (painful crises) and may ameliorate the adverse effect of sickle cell anemia on the liver. It is also suggested that one of the antisickling effects or mechanisms of action of Ciklavit may involve the induction of fetal hemoglobin production. Ciklavit may therefore be a promising option for the treatment and management of sickle cell anemia. 

## 7. Current Research Methodologies on Antisickling Phytomedicines

### 7.1. *In Vitro* Screening of Plant Samples for Phytochemical, Nutrient, and Antioxidant Composition

A study was carried out to screen the leaf extracts of *Parquetina nigrescens* and *Carica papaya* L. (Caricaceae) for possible antioxidant phytochemicals, proximate nutrient constituents, amino acid composition, and mineral content present in the samples using standard chemical and chromatographic procedures. Phytochemical screening confirmed the presence of folic acid, vitamin B_12_, alkaloids, spooning, glycosides, tannins, and anthraquinones [78]. The study also showed that each of these plant extracts contained flavonoids and the antioxidant vitamins A and C. Some of the previously established antisickling amino acids were also present in the plants. Cyanogenic glycosides were absent from both plant extracts, indicative of the nontoxic effects of these plants when taken orally. These results indicate that the previously reported antisickling properties of these herbs may be due to their inherent antioxidant nutrient composition, thus supporting the claims of the traditional healers and suggesting a possible correlation between the chemical composition of these plants and their uses in traditional medicine [78].

### 7.2. Bioassay Studies on Therapeutic Efficacy and Safety Profile of Selected Plants in Rat Models

#### 7.2.1. Antisickling Potency of Selected Phytomedicines

In a study [53], antisickling data was obtained from three typical independent experiments performed in duplicate using blood samples from twenty SS patients. Sickle cell suspensions were preincubated with extracts prior to exposure to 2% sodium metabisulphite solution. Results showed that the time course for 60% sickling was 40 minutes for the control (SS blood without extract). However, at the same time, PHBA (parahydroxy benzoic acid), saline, Ciklavit, *P. nigrescens,* and *C. papaya *(5 mg/mL) all reduced sickling to 50%, 15%, 5%, 2%, and 0% respectively (Figure 7). *C. papaya* showed the highest antisickling activity after 40 minutes of incubation compared to the other phytomedicines and chemical standards used.

#### 7.2.2. Hb^SS^ Polymerization and Time Course for Sickling

Data from *in vitro* studies on the Time course for antisickling activity of the herbal extracts carried out on blood samples collected from confirmed noncrisis sickle cell individuals showed that all the extracts reflected the same delay time for Hb^SS^ polymerization. 

*Carica papaya* did not prolong the delay time of Hb polymerization but greatly affected (appreciably) the time course for sickling (the most effective dose range being 5–10 mg/mL aqueous extract) compared with *Fagara*, para-hydroxybenzoic acid, *Parquetina nigrescens,* and Ciklavit (Figure 7). The aqueous papaya extracts reduced the degree of sickle cell formation in a dose-dependent manner (1, 3, 5 mg/mL–10 mg/mL), with the highest dose exhibiting a more effective antisickling activity compared to the methanol extract concentrations and the Hb^SS^-sodium metabisulphite control. After 2 hours, in the presence of 5 mg/mL and 10 mg/mL extract concentrations and under the view of the microscope, the original discoidal shape was retained in nearly all cells, unlike the control where over 80% of the SS cells had assumed a sickle shape.

In conclusion, research into phytotherapy of diseases is a current trend in the management of tropical diseases and genetic disorders like sickle cell anemia, with a view to finding cheaper, alternative medicines that the wide populace can have immediate access to. The results outlined in this paper, indicate the feasibility of botanicals, mainly antisickling *phytomedicines* and *nutraceuticals,* as attractive potential candidates for sickle cell anemia therapy and strongly collaborate the ethnomedical usage of the plants.

Further in-depth *in vivo* studies using transgenic mice models and cell lines will provide the mechanism of action and subsequently a deeper appreciation of these phytomedicines and nutraceuticals currently used to improve the quality of life of individuals with sickle cell anemia.

## Abbreviations

SS or Hb^SS^: Hemoglobin S
RBC: Red blood cells.
